# General Commentary: Rethinking the role of animals in human well-being

**DOI:** 10.3389/fpsyg.2013.00374

**Published:** 2013-06-25

**Authors:** Mark A. Oyama, James A. Serpell

**Affiliations:** ^1^Department of Clinical Studies - Philadelphia, Section of Cardiology, University of PennsylvaniaPhiladelphia, PA, USA; ^2^Department of Clinical Studies - Philadelphia, Center for the Interaction of Animals and Society, University of PennsylvaniaPhiladelphia, PA, USA

Recent reports highlight the emerging idea that ownership of companion animals can significantly affect human health: Two articles in Frontiers in Psychology report that dogs provided superior social support to children with disorganized attachment patterns than did other humans (Beetz et al., 2012a), and that the neuropeptide hormone oxytocin is likely involved in the formation of these human-animal bonds and their ability to protect or buffer us from the debilitating effects of stress (Beetz et al., 2012b). In 2013, the American Heart Association endorsed a Scientific Statement on pet ownership and cardiovascular disease risk, stating that pet ownership, and in particular that of dogs, should be considered as a means to reduce cardiovascular risk factors and improve survival in individuals with existing cardiovascular disease (Levine et al., 2013).

The belief that animals could influence human well-being is one that extends back thousands of years to when shamans or “witch doctors” could be called upon to induce either sickness or healing through the evocation of animal “guardian spirits”. Since the late eighteenth century, companion animals have been used as agents of rehabilitation to treat social ostracism, loneliness, and recidivism of the mentally ill, chronically infirmed, and criminal, respectively. An expert none other than Florence Nightingale once remarked that a pet “is often an excellent companion for the sick, for the long chronic cases especially” (Nightingale, 1860). Thus, it could be said that humans have always suspected an ability of animals to improve the human condition. This premise has been applied in a wide variety of conditions including pervasive developmental and psychiatric disorders, victims of sexual abuse, dementia, fibromyalgia, and stroke (Dietz et al., 2012; Muñoz Lasa et al., 2013). The therapeutic potential in these and other disease conditions should not be overlooked.

There are more than 43 million households in the US that own one or more dogs and over half of US households own some species of pet (American Veterinary Medical Association, 2012). Sixty-three percent of these households regard their pets as full-fledged family members. Thus, pets, and the dog in particular, are ubiquitous members of our social environment. It is well-established that social support systems benefit patients with chronic diseases, and whether dogs can either fulfill or enhance this support structure merits additional study. We have previously hypothesized that the “salutary effect of social support should apply to any positive social relationship; any relationship in which a person feels cared for, loved, or esteemed” (Serpell, 2000). Despite the growing evidence that companion animals may contribute socially to human well-being, this field of investigation has still received very limited medical recognition. In 2011 consumers in the US spent over $230 billion dollars for prescription drugs (National Health Expenditure Account, 2011). An equivalent cost-benefit analysis of pet ownership to improve well-being merits closer consideration. In the past, animals have been regarded as a test bed for new therapies or as *models* of human disease. We would encourage consideration of companion animals as *therapy* for disease. This premise deserves serious consideration by the mainstream medical community and research funding agencies.
